# Supplementary material for: Giant absorption of light by molecular vibrations on a chip
Source: Sci Rep. 2016 Feb 18;6:21201. doi: 10.1038/srep21201 (PMC4758027; doi:10.1038/srep21201)
Supplement: Supplementary Information [file srep21201-s1.pdf]

Giant absorption of light by molecular vibrations on a chip: Supplementary Information

A. Karabchevsky,[1,*] and A. V. Kavokin,[2,3*]

[1]Department of Electrooptic Engineering, Ben-Gurion University, 84105, IL

[2]Department of Physics and Astronomy, University of Southampton, SO17 1BJ, UK

[3]CNR-SPIN, Viale del Politecnico 1, I-00133 Rome, Italy

1. Absorption dependence on the pathlength

To check the absorption dependence on the pathlength of light we have dripped the mixture of 67% NMA in Hex onto the surface of the waveguide at two different pathlengths. The recorded spectra at pathlength L=1.5 mm and the doubled pathlength of L=3 mm on a waveguide are shown in Figure 1. We observe a superlinear dependence of the logarithm of transmittance on the length of the waveguide. This is a smoking gun for the diffusive propagation of light in the presence of strong scatters such as NMA molecules.

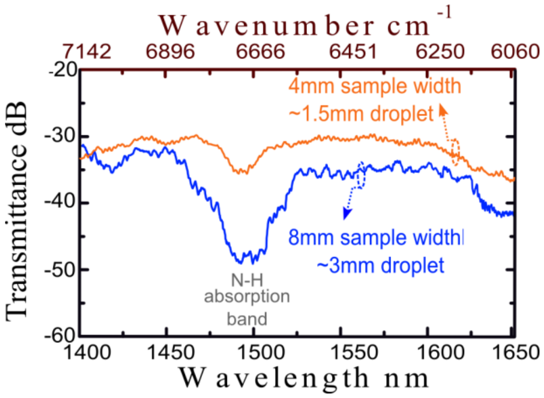

Supplementary Figure 1| Absorption dependence on the pathlength. The spectra have been recorded on a waveguide with L=1.5 mm and L=3 mm distances of the final droplet using 67% NMA in the Hex mixture

2. Beer-Lambert law in the diffusion regime

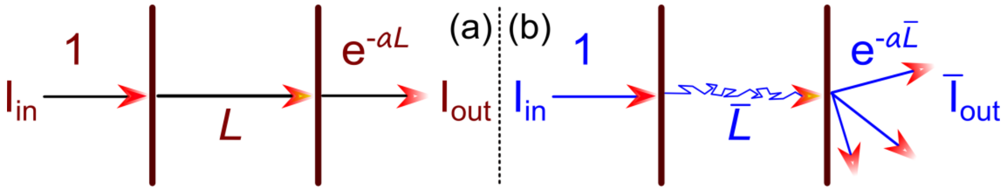

Supplementary Figure 2| Beer-Lambert law in the diffusion regime. a, Incident light I_{in} becomes weaker by e(-αL) as it passes through the solution layer of the width L in the ballistic regime and **b,** it becomes weaker by e(-$\alpha\bar{L}$) in the diffusive regime.

2.1 Relationship between the time and absorption coefficient

In the ballistic regime, the time *t* which a photon spends in the solution is proportional to the distance x:

$$t \propto x \quad \text{(SE1)}$$

while in the diffusion regime, there is a quadratic dependence between *t* and the distance:

$$t \propto x^2 \quad \text{(SE2)}$$

2.2 Relationship between the penetration length and intensity

In the ballistic regime, the dependence of the penetration length of light in the absorbing medium *L* and the output intensity I is: (see Fig. 1(a))

$$I_{out} = e^{-\alpha L} \quad , \quad \text{(SE3)}$$

$$log(I_{out}) = -\alpha L \quad , \quad \text{(SE4)}$$

where $\alpha = \varepsilon c$ with the molar absorptivity ε and the concentration of the compound in the solution c.

In the diffusive regime (see Fig. 1(b)), SE3 and SE4 hold with the length *L* being replaced by an effective length which can be estimated as:

$$\tilde{L} = v\frac{L^2}{D}, \quad \text{(SE5)}$$

where *D* is the diffusion coefficient, v is the ballistic speed of light in the solution.

3. Structured multilayer

1) NIR measurements from cuvettes (conventional spectroscopy) of different NMA/hexane mixtures shown in Figure 1a in the main paper illustrate that the presence of hexane in the mixture shifts the N-H peak towards shorter wavelengths [1]. NIR data recorded from the droplet of 67% NMA diluted in Hex shows that in 35 minutes the centre of the N-H peak shifts from about 1.475 μm to about 1.49 μm, indicating that the concentration of NMA in the region of solution that is sampled near the surface, rises from 4.2% to 100%. This signal then remains steady, demonstrating that the solvent has been excluded from the region near the surface and a stable layer has been formed.

2) Surface tension analysis

   1 μL of water was dispensed onto the surface of K+ exchanged silicate glass that had been treated with oxygen plasma and one that had been both treated with oxygen plasma and immersed in a 67% NMA in hexane mixture for 1 hour at room temperature. Contact angles were measured with a Kruss DSA100 (Figure 4c in the main paper). In average, 4 drops of water were introduced in different areas of each sample. The mean values were reproducible to within $\pm 2°$ of the value reported. Immersion in the solution clearly

induces a chemical change on the surface inducing the increase in its hydrophobicity (see Figure 4b in the paper). The measured contact angles of ∠56° are characteristic of a well packed unprotonated amine terminated layer on glass [2] or a patchy methyl terminated layer on glass [3]. Note: Contact angle measurements recorded from the silicate glass and a K+ exchanged silicate waveguide that were treated with oxygen plasma and immersed in a 67% NMA in hexane mixture for 1 hour at room temperature showed similar results. Whilst measurements were repeated on samples which had been cleaned with solvent but not exposed to oxygen plasma no such change in contact angle occurred, indicating that the oxygen plasma treatment is key to the surface modification process.

3) Ellipsometry

We have measured the thickness of the created layer on our device using Uvisel 2 HORIBA Scientific ellipsometer operating with a 190-880 nm Twin PMT detector. An angle of incidence of 70° from the normal direction to the surface was employed to measure the thickness of the layer from the planar K+ exchanged silicate waveguide immersed in the 67% NMA in Hex mixture. A refractive index of 1.571 at 589 nm was assumed for the film yielding a thickness of 8 nm.

4) Length of the NMA molecule:

Calculated end-to end length of NMA with Spartan 14 is 7.239 Å. Reported values for benzene based adsorbed monolayers are no more than 9Å [2]. From this we conclude that a multilayer structure was formed on the surface of our device.

5) NIR spectroscopy of the dry substrate:

After the droplet has been removed at the end of the experiment the dry film gives no NIR signal. This infers that although the attached surface layer must be present for the optical enhancement to occur either a secondary architecture must extend beyond it (Figure 4a in the main paper) into solution that is washed away when the droplet is removed, or it is the combination of the attached layer and NMA/hexane mixture that causes the optical enhancement.

6) Droplet NIR spectroscopy of 67% Toluene/hexane and 67% Aniline/hexane mixtures were also performed on an identical waveguide. Toluene (index of $n_{Toluene}$=1.4968) and Aniline (index of n_{ANL}=1.5863) share the same basic structure as NMA (index of n_{NMA}=1.571), but the N-methyl group ($N(CH_3)H$) on the benzene ring is replaced with a CH_3 and an NH_2 group respectively. The spectral responses of these mixtures are shown in Figure 4d. No signal enhancement is observed from Toluene, but several trends may be observed by comparison of the spectra from Aniline and NMA. Note: indices of the

liquids were measured using RA 510 refractometer operating at 589 nm at room temperature of 21±2°C.

Although, the measured N-H overtone absorption band of Aniline in the region $\Delta V=2$ (see Figure 1a in the main paper) generally yields a stronger absorption compared to NMA, its absorption is weaker than NMA in our experiments on oxygen plasma treated waveguides.

After 20 minutes on the waveguide the signal from Aniline stabilises, but the N-H peak does not match the wavelength of 1.5 μm recorded from pure Aniline in a cuvette, settling instead at 1.475 μm. Solvent exclusion obviously is not as effective in this mixture, and the concentration of molecules near the surface is likely to be reduced compared to one observed with NMA.

The level of the background loss observed in Figure 4c, is greatly enhanced in Aniline (45 dB) with respect to NMA (30 dB), a phenomenon reported for Aniline hexane mixtures and referred to as critical opalescence [4, 5].

The spectra taken from both Aniline and NMA exhibit broadenings of the N-H overtone peaks, associated with the increased hydrogen bonding to the N-H bond, although this effect is much stronger in Aniline. Weak hydrogen bonding appears to reduce the anharmonicity of the vibration resulting in the change of the overtone absorption peak width [6].

The oxygen plasma treated substrate is strongly hydrophilic and hexane is a very hydrophobic solvent. Once the droplet of hexane / NMA mixture is placed onto the substrate a strong driving force will act to separate the hexane from the substrate. Both NMA and Aniline have a polar hydrophilic head group ($N(CH_3)H$, NH_2) attached to a nonpolar hydrophobic component (the benzene ring) which can act as a separator between the surface and the solvent reducing the surface tension effects, if aligned correctly. Additionally, both molecules contain benzene rings which will be further stabilised if the molecules pack closely with many rings aligned. Hydrogen bonding between aniline molecules is much stronger than in NMA making it less energetically favourable for them to reorganise and align with the surface [7].

Following the monolayer assembly, since benzene rings align in a π stack away from polar media and hydrogen bonding is much stronger in aromatic amines than their linear counterparts, [8] subsequent layers of NMA may align on the surface, forming a structure much like a lamellar liquid crystal in solution [9] some of which would remain when the solvent is removed.

References

[1] Durocher, G., Sandorfy, C. A study of the effects of solvent on overtone frequencies. *J. Mol. Spectrosc.* **22**, 347-359 (1967).

[2] Barriet, D., Yam, C. M., Shmakova, O. E., Jamison, A. C., Lee, T. R. 4-Mercaptophenylboronic Acid SAMs on gold: comparison with SAMs derived from thiophenol, 4-mercaptophenol, and 4- mercaptobenzoic Acid. *Langmuir* **23**, 8866-8875 (2007).

[3] Peters, R. D., Yang, X. M., Kim, T. K., Sohn, B. H., Nealey, P. F. Using Self-Assembled monolayers exposed to X-rays to control the wetting behaviour of thin films of diblock copolymers. *Langmuir* **16**, 4625-4631 (2000).

[4] Snyder, R. B., Eckert C.A. Effect of third component on liquid-liquid critical-point. *J. Chem. Eng. Data* **18**, 282-285 (1973)

[5] Alonso-Tristan, Gonzalez, C., J. A., Fuente, I. G. de la, Cobos, J. C. Thermodynamics of mixtures containing amines. XV. Liquid−Liquid equilibria for benzylamine + $CH_3(CH_2)_nCH_3$ (n = 8, 9, 10, 12, 14). *J. Chem. Eng. Data* **59**, 2101-2105 (2014).

[6] Vollhardt, K. P. C., Shore, N. E. Organic Chemistry. 2nd edition, (Freeman, New York) 594-628 (1948).

[7] Mardis, K. L., Brune, B. J., Vishwanath, P., Giorgis, B., Payne, G. F., Gilson, M. K. Intramolecular versus intermolecular hydrogen bonding in the adsorption of aromatic alcohols onto an acrylic ester sorbent. *J. Phys. Chem. B* **104**, 4735-4744 (2000).

[8] Bernard-Houplain, M. C., Belanger, G., Sandorfy, C., Low temperature infrared of Association in dissolved N-Methylaniline. *J. Chem. Phys*. **57,** 530–533 (1972).

[9] Atkins, P. W., Physical Chemistry. 5th edition, (Oxford university press) 972-973 (1994).
